# 2380. Menstrual changes after COVID vaccine in the Dominican Republic

**DOI:** 10.1093/ofid/ofad500.2001

**Published:** 2023-11-27

**Authors:** Yori A Roque, David De Luna

**Affiliations:** Hospital Metropolitano de Santiago (HOMS), Santiago, Santiago, Dominican Republic; Hospital Metropolitano de Santiago, Santiago, Santiago, Dominican Republic

## Abstract

**Background:**

Women around the world began reporting menstrual disorders after receiving COVID vaccines. Numerous studies were launched evaluating a possible causal relationship between vaccines and these disorders. To date, it has not been possible to establish causality between the different vaccine compounds and these disorders. Here we identify the main self-reported menstrual disorders in vaccinated women the Dominican Republic.

**Methods:**

We elaborated a multiple-choice self-questionnaire shared through a link on social networks. Sociodemographic factors, type of vaccine received, number of doses, and symptoms or disorders related to the menstrual cycle that occurred after its administration were addressed.

**Results:**

200 participants completed the questionnaire. The ages were mainly between 18 to 28 years (40.5%), 29 to 38 years (39%), 39 to 49 years (18.5%). 74% defined their menstrual cycle as regular (between 21 and 29 days). Most of the vaccines reported were Coronavac (63%), Astra Zeneca (23%) and Pfizer (7%). 81.5% received two doses of the vaccine, while 13.5% had received 3 doses. Most of the disorders reported occurred after the second dose (60%), the most frequent being dysmenorrhea (33%), menorrhagia (31.5%) delayed menstrual bleeding (31%), and heavy bleeding (30.5%). 59.5% considered these symptoms as moderate and 44.5% reported that they were maintained for 2 to 3 months. 65.5% of women reported that they would get vaccinated against COVID again despite these events.

**Figure:**
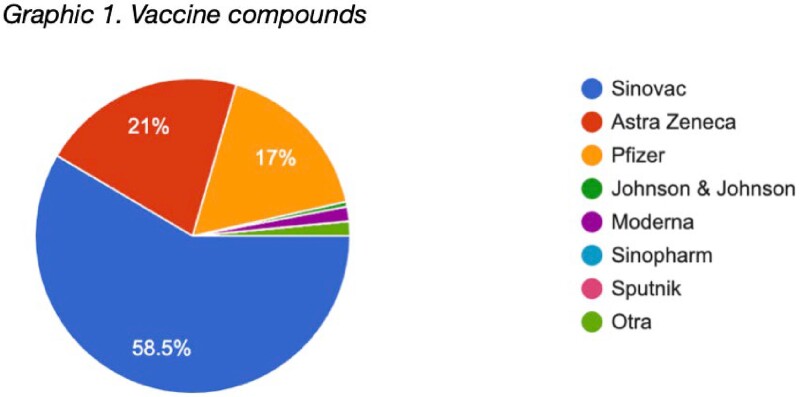

**Figure:**
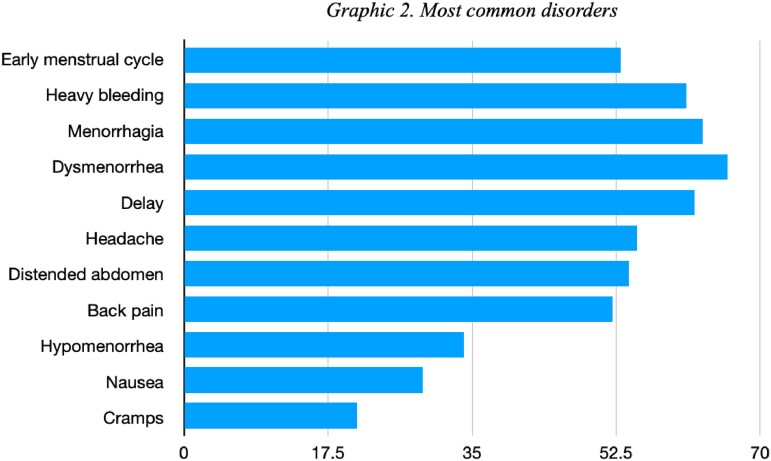

**Figure:**
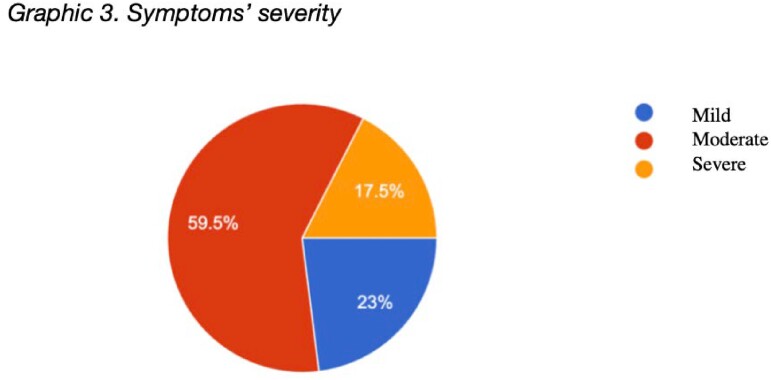

**Conclusion:**

Dysmenorrhea, menorrhagia, delayed menstrual bleeding, and heavy bleeding were the most reported disorders in our cohort. Although causality has not been confirmed, health personnel should be aware of these findings and offer the relevant recommendations to women who are going to be vaccinated.

**Disclosures:**

**All Authors**: No reported disclosures

